# Sub-Geometric Phases in Density Matrices

**DOI:** 10.1038/s41598-019-49770-1

**Published:** 2019-09-13

**Authors:** Zheng-Chuan Wang

**Affiliations:** Department of Physics & CAS Center for Excellence in Topological, Quantum Computation, The University of Chinese Academy of Sciences, P. O. Box 4588, Beijing, 100049 China

**Keywords:** Quantum mechanics, Statistical physics

## Abstract

This study presents the generalization of geometric phases in density matrices. We show that the extended sub-geometric phase has an unified expression during the adiabatic or nonadiabatic process and establish the relations between them and the usual Berry or Aharonov-Anandan phases. We also demonstrate the influence of sub-geometric phases on the physical observables. Finally, the above treatment is used to investigate the geometric phase in a mixed state.

## Introduction

Geometric phase^1,2^, since its discovery in 1984, had been widely explored in theories and experiments^3^. Nowadays, it plays an important role in many fields^4^, such as condensed matter physics, atomic and molecular physics, optics, quantum field and topological physics. Generally, geometric phase was investigated by the Schrödinger equation. However, it can also be studied by the path integral formalism^5^ and relativistic Dirac equation^6^. Herein, a different method was used. We focus attention on the density matrix of the system, rather than on the wave function. The usual geometric phase, both the Berry phase^1^ in the adiabatic evolution of wave function $|{\rm{\psi }}({\rm{t}})\rangle ={{\rm{e}}}^{{\rm{i}}{\int }_{R(0)}^{R(t)}i\langle {\psi }_{n}(R)|{\nabla }_{R}|{\psi }_{n}(R)\rangle dR}{e}^{-\frac{i}{\hslash }{\int }_{0}^{t}{E}_{n}(R(t^{\prime} ))dt^{\prime} }|{\psi }_{n}(R(t))\rangle$ with instantaneous eigenstate $|{\psi }_{n}(R(t))\rangle$ and eigen energy ${E}_{n}(R(t))$, and the Aharonov-Anandan (AA) phase^7^ in the nonadiabatic evolution of wave function $|{\rm{\psi }}({\rm{t}})\rangle ={{\rm{e}}}^{{\rm{i}}{\int }_{0}^{t}i\langle \tilde{\psi }(t^{\prime} )|\frac{\partial }{\partial t^{\prime} }|\tilde{\psi }(t^{\prime} )\rangle dt^{\prime} }{e}^{-\frac{i}{\hslash }{\int }_{0}^{t}\langle \psi (t^{\prime} )|H(t^{\prime} )|\psi (t^{\prime} )\rangle dt^{\prime} }|\tilde{\psi }(t)\rangle$ with Hamiltonian $H(R(t))$, are canceled by their conjugate and therefore do not contribute to their density matrices. This motivates us to find a geometric phase that can appear in the density matrix and allows us to address the effects of geometric phase on the physical observables.

The geometric phase in the wave function is usually observed via interference experiments. Does the geometric phase produce other observable effects? Several works had been done toward this goal. In 1983, Thouless *et al*. demonstrated the influence of geometric phase on the adiabatic particle transport process^8^. They also elaborated the quantized Hall conductance via geometric phase^9^. Based on the path integral formalism, Huratsuji *et al*. suggested that the geometric phase may affect the quantization rule^5^ and hence the quantized energy. To illuminate the influence of geometric phase on the acoustoelectric current induced by surface acoustoelectric waves, we developed a position dependent geometric phase, and exploited it to study this quantized acoustoelectric current^10^. Further, can we expect this geometric phase to have effect on other physical observables? To our knowledge, this is an open question and worth to explore. The usual geometric phases do not appear in the density matrices; however, the physical observables are always calculated via the statistical average by the density matrix. Thus, the geometric phase that can exist in the density matrix must be determined.

## The Sub-Geometric Phases

Initially, we studied the adiabatic evolution of a system $H(R(t))$ with parameter $R(t)$, whose eigenequation is $H(R(t))|{\psi }_{n}(R(t))\rangle ={E}_{n}(R(t))|{\psi }_{n}(R(t))\rangle$. To obtain the Berry phase, the instantaneous eigenstate $|{\psi }_{n}(R(t))\rangle$ should be calculated. However, it is seem not trivial because the instantaneous eigenequation is difficult to solve except in some special cases, which is discussed below.

If we denote the Hamiltonian at initial time as $H(R(0))$ with its eigenequation $H(R(0))|{\psi }_{n}^{0}\rangle ={E}_{n}^{0}{\psi }_{n}^{0}$, the time-dependent Hamiltonian can be divided as follows:

1 $$H(R(t))=H(R(0))+\Delta H(R(t)),$$

where $\Delta H(R(t))\,$describes the difference between the Hamiltonian $H(R(t))$ and its initial value $H(R(0))$, which is usually small when the system varies adiabatically. We can now perform the expansion of the instantaneous eigenfunction by the complete basis $\{|{\psi }_{n}^{0}\rangle \}$ of $H(R(0))$ as follows:

2 $$|{\psi }_{n}(R(t))\rangle =\sum _{k}\,{c}_{nk}(R(t)){e}^{-i{\omega }_{k}t}|{\psi }_{k}^{0}\rangle ,$$

where ${\omega }_{k}=\frac{{E}_{k}}{\hslash }$, and the coefficient ${c}_{nk}(R(t))$ satisfies

3 $${\rm{i}}\hslash {\dot{c}}_{nk}(R(t))=\sum _{l}\,{e}^{-i{\omega }_{kl}t}\langle {\psi }_{k}^{0}|\Delta H(R(t))|{\psi }_{l}^{0}\rangle \,{c}_{nl},$$

with ${\omega }_{kl}=\frac{{E}_{k}}{\hslash }-\frac{{E}_{l}}{\hslash }$. In the case of adiabatic evolution, $\Delta H(R(t))$ may be regarded as perturbation, therefore, ${c}_{nk}$ may take the form of the first-order approximation as follows: ${c}_{nk}(R(t))=\frac{1}{{\rm{i}}\hslash }\,{\int }_{0}^{t}\,{e}^{-i{\omega }_{nk}t^{\prime} }\langle {\psi }_{k}^{0}|\Delta H(R(t^{\prime} ))|{\psi }_{k}^{0}\rangle dt^{\prime}$. It should be ponied out that Eqs (1–3) are still suitable for the description of nonadiabatic case, in which we can not solve Eq. (3) by the perturbative theory again.

Considering Eq. (2), if we introduce a notation such that $|{\phi }_{nk}(R(t))\rangle$ represents the basis ${c}_{nk}(R(t))|{\psi }_{k}^{0}\rangle$ and let ${e}^{if(t)}{e}^{-i{\omega }_{k}t}|{\phi }_{nk}(R(t))\rangle \,$ satisfy the Schrödinger equation, ${\rm{f}}({\rm{t}})$ can be solved as follows:

4 $$\begin{array}{rcl}{\rm{f}}({\rm{t}}) & = & {\rm{i}}\,{\int }_{R(0)}^{R(t)}\,\frac{\langle {\phi }_{nk}(R)|{\nabla }_{R}|{\phi }_{nk}(R)\rangle dR}{\langle {\phi }_{nk}(R)|{\phi }_{nk}(R)\rangle }\\  &  & -\,\frac{1}{\hslash }\,{\int }_{0}^{t}\,\frac{\langle {\phi }_{nk}(R(t^{\prime} ))|\Delta H(R(t^{\prime} ))|{\phi }_{nk}(R(t^{\prime} ))\rangle dt^{\prime} }{\langle {\phi }_{nk}(R(t^{\prime} ))|{\phi }_{nk}(R(t^{\prime} ))\rangle }.\end{array}$$

Now $\{{e}^{if(t)}{e}^{-i{\omega }_{k}t}|{\phi }_{nk}(R(t))\rangle \}$ constitutes a complete basis. The instantaneous eigenfunction can also expanded as follows:

5 $$\begin{array}{rcl}|{\psi }_{n}(R(t))\rangle  & = & \sum _{k}\,{a}_{nk}\,{e}^{{\rm{i}}{\int }_{R(0)}^{R(t)}\frac{i\langle {\phi }_{nk}(R)|{\nabla }_{R}|{\phi }_{nk}(R)\rangle dR}{\langle {\phi }_{nk}(R)|{\phi }_{nk}(R)\rangle }}\\  &  & \,{e}^{-i({\omega }_{k}t+\frac{1}{\hslash }{\int }_{0}^{t}\frac{\langle {\phi }_{nk}(R(t^{\prime} ))|\Delta H(R(t^{\prime} ))|{\phi }_{nk}(R(t^{\prime} ))\rangle  > dt^{\prime} }{\langle {\phi }_{nk}(R(t^{\prime} ))|{\phi }_{nk}(R(t^{\prime} ))\rangle })}{c}_{nk}(R(t))|{\psi }_{k}^{0}\rangle ,\end{array}$$

where the coefficients $\{{a}_{nk}\}$ can be absorbed into $|{\phi }_{nk}(R(t))\rangle$, we will not write them out in the following. The phase ${\int }_{R(0)}^{R(t)}\,\frac{i\langle {\phi }_{nk}(R)|{\nabla }_{R}|{\phi }_{nk}(R)\rangle dR}{\langle {\phi }_{nk}(R)|{\phi }_{nk}(R)\rangle }$ in the above expression is just the geometric phase associated with the basis $|{\phi }_{nk}(R(t))\rangle ={c}_{nk}(R(t))|{\psi }_{k}^{0}\rangle$ and is named as the sub-geometric phase, whereas the phase ${\omega }_{k}t+\frac{1}{\hslash }\,{\int }_{0}^{t}\,\frac{\langle {\phi }_{nk}(R(t^{\prime} ))|\Delta H(R(t^{\prime} ))|{\phi }_{nk}(R(t^{\prime} ))\rangle dt^{\prime} }{\langle {\phi }_{nk}(R(t^{\prime} ))|{\phi }_{nk}(R(t^{\prime} ))\rangle }$ is the dynamical phase. Following Eq. (5), its corresponding density matrix can be represented as follows:

6 $$\begin{array}{rcl}{\rho }_{n} & = & \sum _{kl}\,{e}^{{\rm{i}}{\int }_{R(0)}^{R(t)}\frac{i\langle {\phi }_{nk}(R)|{\nabla }_{R}|{\phi }_{nk}(R)\rangle dR}{\langle {\phi }_{nk}(R)|{\phi }_{nk}(R)\rangle }-{\int }_{R(0)}^{R(t)}\frac{i\langle {\phi }_{nl}(R)|{\nabla }_{R}|{\phi }_{nl}(R)\rangle dR}{\langle {\phi }_{nl}(R)|{\phi }_{nl}(R)\rangle }}\\  &  & \,{e}^{-i({\omega }_{kl}t+\frac{1}{\hslash }{\int }_{0}^{t}\frac{\langle {\phi }_{nk}(R(t^{\prime} ))|\Delta H(R(t^{\prime} ))|{\phi }_{nk}(R(t^{\prime} ))\rangle dt^{\prime} }{\langle {\phi }_{nk}(R(t^{\prime} ))|{\phi }_{nk}(R(t^{\prime} ))\rangle }-\frac{1}{\hslash }{\int }_{0}^{t}\frac{\langle {\phi }_{nl}(R(t^{\prime} ))|\Delta H(R(t^{\prime} ))|{\phi }_{nl}(R(t^{\prime} ))\rangle dt^{\prime} }{\langle {\phi }_{nl}(R(t^{\prime} ))|{\phi }_{nl}(R(t^{\prime} ))\rangle })}\\  &  & \,{c}_{nl}^{\ast }(R(t)){c}_{nk}(R(t))|{\psi }_{k}^{0}\rangle \langle {\psi }_{l}^{0}|.\end{array}$$

The phase ${\int }_{R(0)}^{R(t)}\,\frac{i\langle {\phi }_{nk}(R)|{\nabla }_{R}|{\phi }_{nk}(R)\rangle dR}{\langle {\phi }_{nk}(R)|{\phi }_{nk}(R)\rangle }$ − ${\int }_{R(0)}^{R(t)}\,\frac{i\langle {\phi }_{nl}(R)|{\nabla }_{R}|{\phi }_{nl}(R)\rangle dR}{\langle {\phi }_{nl}(R)|{\phi }_{nl}(R)\rangle }$ is just the relative sub-geometric phase between states $|{\psi }_{k}^{0}\rangle$ and $|{\psi }_{l}^{0}\rangle$ in the density matrix.

Using Eq. (2), the sub-geometric phase can be connected with the usual Berry phase, which can be expressed as ${\rm{i}}\,{\int }_{R(0)}^{R(t)}\,\langle {\psi }_{n}(R)|{\nabla }_{R}|{\psi }_{n}(R)\rangle dR$ = ${\rm{i}}\,{\int }_{R(0)}^{R(t)}\,{\sum }_{k}\,{c}_{nk}^{\ast }(R(t))\frac{\partial }{\partial R}{c}_{nk}(R(t))dR$, and the sub-geometric phase is ${\rm{i}}\,{\int }_{R(0)}^{R(t)}\,\frac{{c}_{nk}^{\ast }(R(t))\frac{\partial }{\partial R}{c}_{nk}(R(t))}{{c}_{nk}^{\ast }(R(t)){c}_{nk}(R(t))}dR$, we find that the Berry phase can be regarded as the summation of sub-geometric phases with the following probability:

7 $${\rm{i}}\,{\int }_{R(0)}^{R(t)}\,\langle {\psi }_{n}(R)|{\nabla }_{R}|{\psi }_{n}(R)\rangle dR={\rm{i}}\,{\int }_{R(0)}^{R(t)}\,\sum _{k}\,|{c}_{nk}{|}^{2}\frac{{c}_{nk}^{\ast }(R(t))\frac{\partial }{\partial R}{c}_{nk}(R(t))}{{c}_{nk}^{\ast }(R(t)){c}_{nk}(R(t))}dR,$$

which relates the Berry phase and the sub-geometric phase proposed herein.

In fact, although the Berry phase will cancel with its conjugate in the expression of density operator when the wavefunction evolves as $|{\rm{\psi }}({\rm{R}}({\rm{t}}))\rangle \cong {{\rm{e}}}^{{\rm{i}}{\int }_{R(0)}^{R(t)}i\langle n(R)|{\nabla }_{R}|n(R)\rangle dR}{e}^{-\frac{i}{\hslash }{\int }_{0}^{t}{E}_{n}(R(t^{\prime} ))dt^{\prime} }|n(R(t))\rangle$ in the adiabatic case, it should be noted that this wavefunction is an approximate form, its exact expression should be

8 $$|{\rm{\psi }}({\rm{R}}({\rm{t}}))\rangle =\sum _{m}\,{a}_{m}\,{{\rm{e}}}^{{\rm{i}}{\int }_{R(0)}^{R(t)}i\langle m(R)|{\nabla }_{R}|m(R)\rangle dR}{e}^{-\frac{i}{\hslash }{\int }_{0}^{t}{E}_{m}(R(t^{\prime} ))dt^{\prime} }|m(R(t))\rangle ,$$

where $\{{{\rm{e}}}^{{\rm{i}}{\int }_{R(0)}^{R(t)}i\langle m(R)|{\nabla }_{R}|m(R)\rangle dR}{e}^{-\frac{i}{\hslash }{\int }_{0}^{t}{E}_{m}(R(t^{\prime} ))dt^{\prime} }|m(R(t))\rangle \}(m=1,2\ldots )$ constitutes a complete basis for expanding the wavefunction $|{\rm{\psi }}({\rm{R}}({\rm{t}}))\rangle$. According to quantum adiabatic theorem, the probability ${|{a}_{m}|}^{2}(m\ne n)$ for the system in other eigenstates except the initial eigenstate $|{\rm{n}}\rangle$ is very small, ${|{a}_{m}|}^{2}\ll 1$. Employing the exact expression (8), one can obtain its density operator as:

9 $$\begin{array}{rcl}{\rm{\rho }}({\rm{R}}({\rm{t}})) & = & \sum _{n,m}\,{a}_{n}(t){a}_{m}^{\ast }(t)\,\exp [\,-\,\frac{i}{\hslash }\,{\int }_{0}^{t}\,({E}_{n}(R(t^{\prime} ))-{E}_{m}(R(t^{\prime} )))dt^{\prime} ]\\  &  & \times \,e{\rm{xp}}[{\rm{i}}\,{\int }_{0}^{t}\,(i\langle n(R(t^{\prime} ))|\frac{\partial }{\partial t^{\prime} }|n(R(t^{\prime} ))\rangle \\  &  & -\,i\langle m(R(t^{\prime} ))|\frac{\partial }{\partial t^{\prime} }|m(R(t^{\prime} ))\rangle )dt^{\prime} ]|n(R(t)\rangle \langle m(R(t))|.\end{array}$$

Equations (8) and (9) are very similar to Eqs (5) and (6) for the sub-geometric phase, they are based on the instantaneous eigenstate representation, while the sub-geometric phases are based on the initial eigenstate representation, but they are the same in the physical nature. It can be seen that the Berry phase $\exp [{\rm{i}}\,{\int }_{0}^{t}\,(i\langle n(R(t^{\prime} ))|\frac{\partial }{\partial t^{\prime} }|n(R(t^{\prime} ))\rangle$ − $i\langle m(R(t^{\prime} ))|\frac{\partial }{\partial t^{\prime} }|m(R(t^{\prime} ))\rangle )dt^{\prime} ]$ shown in expression (9) is the relative Berry phase between the instantaneous eigenstates $|{\rm{n}}(R(t^{\prime} ))\rangle$ and $|m(R(t^{\prime} ))\rangle$, they appear only in the off-diagonal elements of the density matrix like the sub-geometric phase.

Compared with the adiabatic evolution, the above sub-geometric phase is suitable for the description of the nonadiabatic case, in which the Hamiltonian ${\rm{H}}({\rm{t}})$ can also be written as ${\rm{H}}({\rm{t}})={H}_{0}+\Delta H(t)$, and its wave function may still be expanded similar to Eq. (5) as follows:

10 $$|\psi (t)\rangle =\sum _{k}\,{e}^{{\rm{i}}{\int }_{0}^{t}\frac{i\langle {\phi }_{k}(t^{\prime} )|\frac{\partial }{\partial t^{\prime} }|{\phi }_{k}(t^{\prime} )\rangle dt^{\prime} }{\langle {\phi }_{k}(t^{\prime} )|{\phi }_{k}(t^{\prime} )\rangle }}{e}^{-i({\omega }_{k}t+\frac{1}{\hslash }{\int }_{0}^{t}\frac{\langle {\phi }_{k}(t^{\prime} )|\Delta H(t^{\prime} )|{\phi }_{k}(t^{\prime} )\rangle dt^{\prime} }{\langle {\phi }_{k}(t^{\prime} )|{\phi }_{k}(t^{\prime} )\rangle })}{c}_{k}(t)|{\psi }_{k}^{0}\rangle ,$$

where ${\int }_{0}^{t}\,\frac{i\langle {\phi }_{k}(t^{\prime} )|\frac{\partial }{\partial t^{\prime} }|{\phi }_{k}(t^{\prime} )\rangle dt^{\prime} }{\langle {\phi }_{k}(t^{\prime} )|{\phi }_{k}(t^{\prime} )\rangle }$ is the sub-geometric phase. We can now see that the expression has the same form as that in the adiabatic case. It should be emphasized that $\Delta H(t)$ is not small in the nonadiabatic procedure; therefore, the coefficients $\{{c}_{k}(t)\}$ in the above expression cannot be obtained via perturbative theory as in the adiabatic case. However, they still satisfy Eq. (3), and we can solve them from these coupled differential equations. Beyond this, the sub-geometric phase in the density matrix is also analogous to the adiabatic case (6), in which the relative geometric phase ${\int }_{0}^{t}\,\frac{i\langle {\phi }_{k}(t^{\prime} )|\frac{\partial }{\partial t^{\prime} }|{\phi }_{k}(t^{\prime} )\rangle dt^{\prime} }{\langle {\phi }_{k}(t^{\prime} )|{\phi }_{k}(t^{\prime} )\rangle }$ − ${\int }_{0}^{t}\,\frac{i\langle {\phi }_{l}(t^{\prime} )|\frac{\partial }{\partial t^{\prime} }|{\phi }_{l}(t^{\prime} )\rangle dt^{\prime} }{\langle {\phi }_{l}(t^{\prime} )|{\phi }_{l}(t^{\prime} )\rangle }$ indicates the coherence between states $|{\psi }_{k}^{0}\rangle$ and $|{\psi }_{l}^{0}\rangle$. Therefore, the sub-geometric phase reported herein has a unified expression during adiabatic and nonadiabatic processes.

Generally, the nonadiabatic AA phase ${\int }_{0}^{t}\,i\langle \tilde{\psi }(t^{\prime} )|\frac{\partial }{\partial t^{\prime} }|\tilde{\psi }(t^{\prime} )\rangle dt^{\prime}$ is not easy to calculate owing to the unknown function $|\tilde{\psi }(t)\rangle$. However, it can be explicitly expressed by the coefficient ${c}_{k}(t)$ in Eq. (2), where the total phase is ${{\rm{e}}}^{{\rm{i}}\varphi }=\langle {\rm{\psi }}(0)|{\rm{\psi }}({\rm{\tau }})\rangle$, yielding $\varphi =-\,i\,\mathrm{ln}\,{\sum }_{n}\,{c}_{n}^{\ast }(0){c}_{n}(\tau ){e}^{-i\frac{{E}_{n}\tau }{\hslash }}$, whereas the dynamical phase is

11 $$\alpha (\tau )=-\,\frac{1}{\hslash }\,{\int }_{0}^{\tau }\,\sum _{n}\,{|{c}_{n}(t)|}^{2}{E}_{n}dt-{\int }_{0}^{\tau }\,dt\,\sum _{nm}\,{c}_{n}^{\ast }(t){c}_{m}(t){e}^{-i\frac{({E}_{m}-{E}_{n})}{\hslash }}\langle {\psi }_{n}^{0}|\frac{\Delta H(t)}{\hslash }|{\psi }_{m}^{0}\rangle .$$

The AA phase β can be finally obtained as ${\rm{\beta }}({\rm{\tau }})=\varphi -{\rm{\alpha }}({\rm{\tau }})$. Therefore, we can provide an alternative way to calculate the AA phase.

Since the sub-geometric phase may appear in the density matrix during adiabatic and nonadiabatic processes, it will affect the physical observables through the statistical average by the density matrix, $\langle {\rm{A}}\rangle ={\rm{tr}}({\rm{\rho }}\hat{A})$. As stated above, the relative sub-geometric phases in the density matrix will not vanish. Instead, they will produce observable effects on these physical quantities, which answers the question raised at the second paragraph.

Employing the sub-geometric phases in the density matrix, we proceed to discuss the geometric phase in a mixed state. For a mixed state ${\rm{\rho }}={\sum }_{k}\,{p}_{k}{\rho }_{k}$, where ${\rho }_{k}$ is the density matrix of the pure state $|{\psi }_{k}\rangle \,(k=1,2\ldots )$ and ${p}_{k}$ is the probability for $|{\psi }_{k}\rangle$ appearing in the mixed state, there is no fixed relative phase including the relative geometric phase between different pure states. Despite some of the treatments proposed^11–17^ for the geometric phase in a mixed state, defining the geometric phase in a mixed state is difficult. Due to the density matrix ${\rho }_{k}$ for a pure state $|{\psi }_{k}\rangle$ comprising the sub-geometric phases, we can naturally define the sub-geometric phases in the density matrix of a mixed state using the proposed method. Certainly, there is still no relative geometric phases between different pure states $\{|{\psi }_{k}\rangle \}$, the sub-geometric phases only exist within each pure state.

## An Example for Two States System

Next, the sub-geometric phase is illustrated using two states system with the Hamiltonian ${\rm{H}}({\rm{t}})={H}_{0}+H^{\prime} (t)$, where

12 $${H}_{0}=(\begin{array}{cc}-\,\Delta  & 0\\ 0 & \Delta \end{array}),\,H^{\prime} (t)=(\begin{array}{cc}0 & W(t)\\ {W}^{\ast }(t) & 0\end{array}),$$

with $W(t)=|W(t)|{e}^{i\delta t}$. For its instantaneous eigenstate $|{\psi }_{-}(t)\rangle =\,\cos \,\frac{\theta }{2}|{\varphi }_{1}\rangle +{e}^{-i\delta }\,\sin \,\frac{\theta }{2}|{\varphi }_{2}\rangle$, where $|{\varphi }_{i}\rangle \,(i=1,2)$ represents the eigenfunction of *H*_0_ and $\tan \,\theta (t)=\frac{|W(t)|}{\Delta }$, the density matrix, including sub-geometric phases is

13 $$\begin{array}{rcl}\rho  & = & co{s}^{2}\frac{\theta }{2}|{\varphi }_{1}\rangle \langle {\varphi }_{1}|\\  &  & +\,{e}^{i({\int }_{0}^{t}\frac{-\frac{1}{2}\sin \frac{\theta }{2}\cos \frac{\theta }{2}\frac{\partial }{\partial t^{\prime} }\theta }{co{s}^{2}\frac{\theta }{2}}dt^{\prime} -{\int }_{0}^{t}\frac{-\frac{1}{2}\sin \frac{\theta }{2}\cos \frac{\theta }{2}\frac{\partial }{\partial t^{\prime} }\theta -si{n}^{2}\frac{\theta }{2}\frac{\partial }{\partial t^{\prime} }\delta }{co{s}^{2}\frac{\theta }{2}}dt^{\prime} )}\,\cos \,\frac{\theta }{2}\,\sin \,\frac{\theta }{2}{e}^{i\delta }|{\varphi }_{1}\rangle \langle {\varphi }_{2}|\\  &  & +\,{e}^{i({\int }_{0}^{t}\frac{\frac{1}{2}\sin \frac{\theta }{2}\cos \frac{\theta }{2}\frac{\partial }{\partial t^{\prime} }\theta -si{n}^{2}\frac{\theta }{2}\frac{\partial }{\partial t^{\prime} }\delta }{si{n}^{2}\frac{\theta }{2}}dt^{\prime} -{\int }_{0}^{t}\frac{-\frac{1}{2}\sin \frac{\theta }{2}\cos \frac{\theta }{2}\frac{\partial }{\partial t^{\prime} }\theta }{co{s}^{2}\frac{\theta }{2}}dt^{\prime} )}\,\cos \,\frac{\theta }{2}\,\sin \,\frac{\theta }{2}{e}^{-i\delta }|{\varphi }_{2}\rangle \langle {\varphi }_{1}|\\  &  & +\,si{n}^{2}\frac{\theta }{2}|{\varphi }_{2}\rangle \langle {\varphi }_{2}|,\end{array}$$

where ${\int }_{0}^{t}\,\frac{-\,\frac{1}{2}\,\sin \,\frac{\theta }{2}\,\cos \,\frac{\theta }{2}\frac{\partial }{\partial t^{\prime} }\theta }{co{s}^{2}\frac{\theta }{2}}dt^{\prime}$ − ${\int }_{0}^{t}\,\frac{-\,\frac{1}{2}\,\sin \,\frac{\theta }{2}\,\cos \,\frac{\theta }{2}\frac{\partial }{\partial t^{\prime} }\theta -si{n}^{2}\frac{\theta }{2}\frac{\partial }{\partial t^{\prime} }\delta }{co{s}^{2}\frac{\theta }{2}}dt^{\prime}$ is the relative sub-geometric phase between states $|{\varphi }_{1}\rangle$ and $|{\varphi }_{2}\rangle$, which is suitable for both adiabatic and nonadiabatic processes. The usual Berry phase is ${\beta }_{Berry}={\int }_{0}^{t}\,si{n}^{2}\frac{\theta }{2}\frac{\partial }{\partial t^{\prime} }\delta dt^{\prime}$, which implies that the Berry phase is the summation of our sub-geometric phases with certain probability. The AA phase ${\beta }_{AA}=\varphi -\alpha (\tau )$ can also be calculated using the proposed method, where $\varphi =-\,{\rm{i}}\,\mathrm{ln}[\cos \,\frac{\theta (0)}{2}\,\cos \,\frac{\theta (\tau )}{2}+{e}^{i(\delta (0)-\delta (\tau ))}\,\sin \,\frac{\theta (0)}{2}\,\sin \,\frac{\theta (\tau )}{2}]$ is the total phase and ${\rm{\alpha }}({\rm{\tau }})=-\,{\int }_{0}^{\tau }\sqrt{{\Delta }^{2}+|W(t){|}^{2}}dt$ is the dynamical phase.

## Experimental Observation of Sub-Geometric Phase

**Figure 1:**
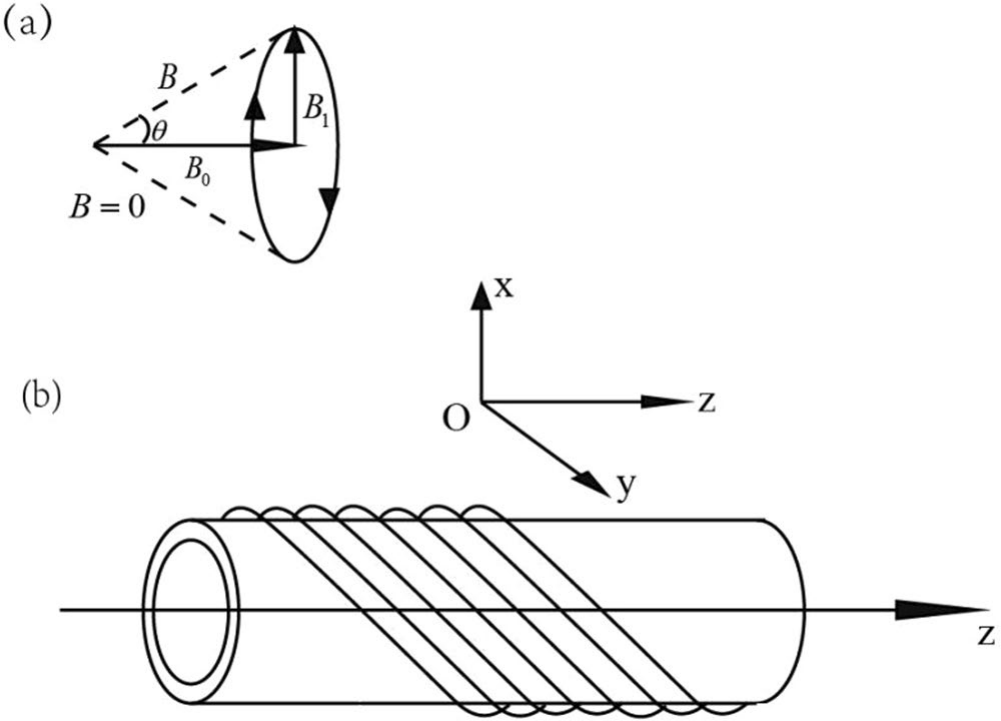
The rotating magnetic field $\overrightarrow{B}(t)$ (**a**) which is produced by the solenoid (**b**).

Consider a spin-1/2 particle under a rotating magnetic field $\overrightarrow{B}(t)=({B}_{1}(t)\,\cos \,2{\omega }_{0}t-{B}_{1}(t)\,\sin \,2{\omega }_{0}t\,{B}_{0})$ which is shown in Fig. 1, where ${\omega }_{0}=\frac{\mu {B}_{0}}{\hslash }$, with $\overrightarrow{\mu }=\mu \sigma$ is the magnetic moment of the particle. When the particle passes through the solenoid, it will experiences a periodic rotating magnetic field $\overrightarrow{{B}_{1}}$(t). The Hamiltonian for this system can be written as

14 $${\rm{H}}({\rm{t}})=-\,\overrightarrow{\mu }\cdot \overrightarrow{B}=(\begin{array}{cc}-\,\mu {B}_{0} & -\,\mu {B}_{1}(t){e}^{2i{\omega }_{0}t}\\ -\,\mu {B}_{1}(t){e}^{-2i{\omega }_{0}t} & \mu {B}_{0}\end{array}).$$

Comparing with Eq. (12), we find that $\Delta =\mu {B}_{0},W(t)=-\,\mu {B}_{1}(t){e}^{2i{\omega }_{0}t}$, $\delta (t)=2{\omega }_{0}t$ and $\tan \,\theta (t)=-\,\frac{{B}_{1}(t)}{{B}_{0}}$. In the next, we also choose the instantaneous eigenstate $$|{\psi }_{-}(t)\rangle =\,\cos \,\frac{\theta }{2}(\begin{array}{c}1\\ 0\end{array})+{e}^{-2i{\omega }_{0}t}\,\sin \,\frac{\theta }{2}(\begin{array}{c}0\\ 1\end{array})$$ to study. According to the notation in Eqs (10) and (13), we have $$|{\phi }_{1}(t)\rangle =\,\cos \,\frac{\theta }{2}(\begin{array}{c}1\\ 0\end{array})$$ and $$|{\phi }_{2}(t)\rangle ={e}^{-2i{\omega }_{0}t}\,\sin \,\frac{\theta }{2}(\begin{array}{c}0\\ 1\end{array})$$ here, then the sub-geometric phase corresponding to $|{\phi }_{1}(t)\rangle$ is ${\gamma }_{1}=-\,\mathrm{ln}|\cos \,\frac{\theta (t)}{2}|+\,\mathrm{ln}|\cos \,\frac{\theta (0)}{2}|$, and the sub-geometric phase to $|{\phi }_{2}(t)\rangle$ is ${\gamma }_{2}=\,\mathrm{ln}|\sin \,\frac{\theta (t)}{2}|-\,\mathrm{ln}|\sin \,\frac{\theta (0)}{2}|$. Similar to Eq. (10), the instantaneous eigenstate with sub-geometric phases can be expressed as

15 $$|{\psi }_{-}(t)\rangle ={e}^{i{\gamma }_{1}}{{\rm{e}}}^{-{\rm{i}}\frac{\mu {B}_{0}t}{\hslash }}\,\cos \,\frac{\theta }{2}(\begin{array}{c}1\\ 0\end{array})+{e}^{i{\gamma }_{2}}{{\rm{e}}}^{{\rm{i}}\frac{\mu {B}_{0}t}{\hslash }}\,\sin \,\frac{\theta }{2}(\begin{array}{c}0\\ 1\end{array}).$$

**Figure 2:**
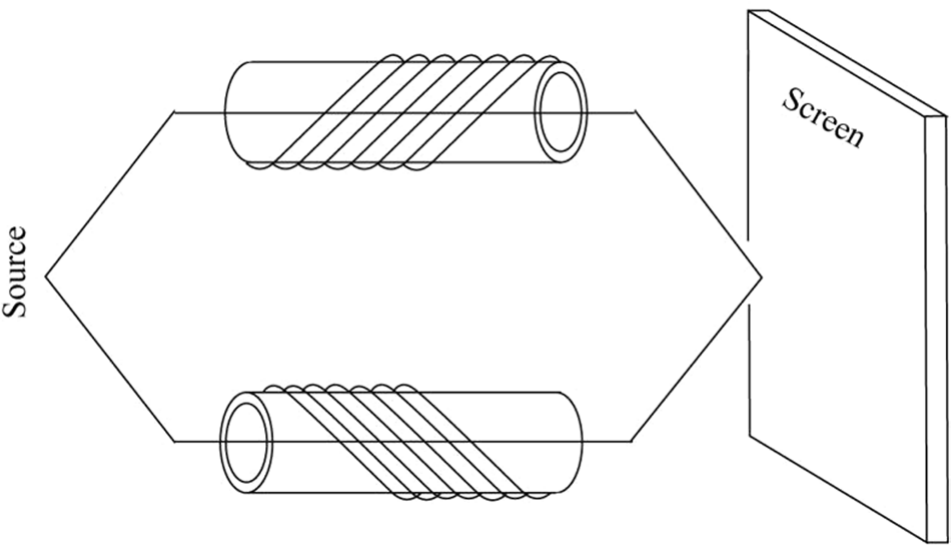
The experimental setup for the interference of two beams of particles with sub-geometric phases which is induced by the rotating magnetic fields produced by the two solenoids with the opposite phase.

In order to observe the sub-geometric phase *γ*_2_ in Eq. (15), we try to devise an experimental setup which is depicted in Fig. 2

The spin-1/2 particles from one source are split into two beams which pass through two solenoids, respectively, then they meet together on the screen and interfere with each other. The magnetic field subject to the two solenoids rotating with the opposite phase, that is ${\overrightarrow{B}}^{1}(t)=({B}_{1}(t)\,\cos \,2{\omega }_{0}t-{B}_{1}(t)\,\sin \,2{\omega }_{0}t\,{B}_{0})$, and ${\overrightarrow{B}}^{2}(t)=({B}_{1}(t)\,\cos \,2{\omega }_{0}t\,{B}_{1}(t)\,\sin \,2{\omega }_{0}t\,{B}_{0})$. It is known that the wavefunction on the screen is superposed as

16 $$|\psi (t)\rangle =|{\psi }_{1-}(t)\rangle +|{\psi }_{2-}(t)\rangle ,$$

where $|{\psi }_{2-}(t)\rangle$ has the opposite sub-geometric phase than $|{\psi }_{1-}(t)\rangle$, then the intensity of interference can be obtained by use of the expression (15) as

17 $$|\psi (t){|}^{2}=4co{s}^{2}{\gamma }_{1}co{s}^{2}\frac{\theta }{2}+4co{s}^{2}{\gamma }_{2}si{n}^{2}\frac{\theta }{2},$$

we can see that the interference is determined by the sub-geometric phases *γ*_1_ and *γ*_2_, so the sub-geometric phase can be observed by measuring the intensity of interference, which indicates that our sub-geometric phase is physical observable.

## Summary and Discussion

As we know that the usual geometric phase, no matter what Berry phase or AA phase, can not appear in the density matrix because it is canceled by its conjugate. To explore the influence of geometric phase on the physical observables, in this paper, we have shown that a generalized sub-geometric phase can exist in the density matrix, therefore have effect on the physical observables averaged by the density matrix. This sub-geometric phase has an unified expression during the adiabatic and nonadiabatic process, it can also be extended to study the geometric phase in a mixed state.

It should be pointed out that the above sub-geometric phase in a mixed state is only investigated at zero temperature. In fact, it can be extended to study a mixed state at finite temperature, i.e., with a density matrix as follows:

18 $$\rho =\frac{1}{Q}\,\sum _{i}\,{e}^{-\beta {E}_{i}}|{\phi }_{i}\rangle \langle {\phi }_{i}|,$$

where *E*_*i*_ and $|{\phi }_{i}\rangle$ are the eigen energy and eigenstate, respectively. ${p}_{i}=\frac{1}{Q}{e}^{-\beta {E}_{i}}$ with $Q={\sum }_{i}\,{e}^{-\beta {E}_{i}}$ and ${\rm{\beta }}=\frac{1}{kT}$ is the probability for a pure state $|{\phi }_{i}\rangle$ appearing in the mixed state. Once the sub-geometric phase in the density matrix ${\rho }_{i}=|{\phi }_{i}\rangle \langle {\phi }_{i}|$ for a pure state $|{\phi }_{i}\rangle$ is determined, we can obtain the geometric phase in the density matrix $\rho =\frac{1}{Q}\,{\sum }_{i}\,{e}^{-\beta {E}_{i}}{\rho }_{i}$ of a mixed state by the summation with probability ${p}_{i}=\frac{1}{Q}{e}^{-\beta {E}_{i}}$, which is similar to Eq. (4) in ref. ^18^.
